# Child–Pugh Stage Predicts Survival in Hospitalized Patients with Decompensated Cirrhosis: A 10-Year Cohort Study

**DOI:** 10.3390/diagnostics16091349

**Published:** 2026-04-29

**Authors:** Ion Dina, Claudia Georgeta Iacobescu, Ioana Valeria Grigorescu, Ion Daniel Baboi, Marian-Vlad Lapadat, Lavinia Alice Bălăceanu

**Affiliations:** 1 Clinical Department 1—Medical Semiology, “Carol Davila” University of Medicine and Pharmacy, 020021 Bucharest, Romania; ion.dina@umfcd.ro (I.D.); alice.balaceanu@umfcd.ro (L.A.B.); 2 Gastroenterology Department, Clinical and Emergency Hospital “Sf. Ioan”, 042122 Bucharest, Romania; iacobescu_clodi@yahoo.com (C.G.I.); ioana-valeria.grigorescu@rez.umfcd.ro (I.V.G.); 3 Internal Medicine Department, Clinical and Emergency Hospital “Sf. Ioan”, 042122 Bucharest, Romania

**Keywords:** liver cirrhosis, hepatic decompensation, Child–Pugh classification, survival prediction

## Abstract

**Background:** Liver cirrhosis, particularly in its decompensated stages, is associated with high short-term mortality among hospitalized patients. Although the prognostic value of the Child–Pugh classification is well established, its independent impact on survival in real-world tertiary emergency settings requires further evaluation. This study aimed to assess the prognostic role of Child–Pugh stage and other clinical factors on short- and mid-term survival in hospitalized cirrhotic patients over a 10-year period. **Methods:** We conducted a retrospective cohort study including 2831 patients hospitalized for liver cirrhosis between 2015 and 2025. Among them, 631 patients with complete Child–Pugh staging were included in the survival analysis. Survival time was defined as the interval between the first hospitalization and the last recorded discharge or in-hospital death. Survival differences were assessed using Kaplan–Meier curves and log-rank tests, while independent predictors of mortality were identified using multivariate Cox proportional hazards regression. A complementary logistic regression model was used to evaluate predictors of mortality as a binary outcome. **Results:** Among the 631 staged patients, 13.5% were classified as Child–Pugh A, 31.9% as Child–Pugh B and 54.7% as Child–Pugh C. In-hospital mortality increased significantly across stages (1.2%, 9.0% and 46.7%, respectively; *p* < 0.001). One-year survival was 98.7% for Child–Pugh A, 83.6% for Child–Pugh B and 40.7% for Child–Pugh C (log-rank *p* < 0.001). In multivariate Coxregression analysis, the strongest predictor of mortality was mixed cirrhosis type (HR = 8.58, 95% CI: 4.81–15.32, *p* < 0.001). Child–Pugh C was also independently associated with a markedly increased mortality risk compared with Child–Pugh A (HR = 25.11, 95% CI: 3.44–183.29, *p* = 0.002). Alcohol-related etiology (HR = 1.81, 95% CI: 1.09–3.01, *p* = 0.023) and age (HR = 1.18 per SD increase, 95% CI: 1.00–1.39, *p* = 0.050) were additionalindependent predictors. The Cox model demonstrated good discrimination (C-statistic ≈ 0.80). In the logistic regression model, mixed cirrhosis type (OR = 13.28, *p* < 0.001) and Child–Pugh stage (OR = 8.66, *p* < 0.001) were the strongest predictors of mortality, while ascites showed an inverse association after adjustment (OR = 0.62, *p* = 0.036). The logistic model showed excellent discrimination (AUC = 0.865). **Conclusions:** Child–Pugh stage remains a strong and independent predictor of survival in hospitalized patients with decompensated cirrhosis. The marked survival gradient across stages, particularly the substantially reduced survival observed in Child–Pugh C patients, highlights thecontinued clinical utility of this simple classification for early risk stratification intertiary emergency hospital settings.

## 1. Introduction

Chronic liver diseases, particularly cirrhosis, represent a major global health burden and are among the leading causes of liver-related mortality worldwide. Liver cirrhosis represents the final stage of chronic liver disease and a major cause of morbidity and mortality worldwide [1,2]. Disease progression from the compensated Child–Pugh A stage to the decompensated B and C stages is of major clinical importance due to the development of severe complications such as hepatorenal syndrome, variceal bleeding, hepatic encephalopathy and spontaneous bacterial peritonitis [3,4,5]. These complications frequently lead to repeated hospitalizations and an increased healthcare burden [6]. Despite advances in medical management, hospitalized patients with decompensated cirrhosis continue to experience high short-term mortality [7,8,9,10]. Accurate risks tratification is therefore essential for guiding clinical decision-making, optimizing resource allocation and identifying patients who may benefit from intensified monitoring or advanced therapeutic interventions. Among the available prognostic tools, the Child–Pugh classification remains one of the most widely used systems for assessing the severity of liver disease [11,12]. Originally developed to predict postoperative mortality in patients undergoing surgery for portal hypertension, the score integrates five clinical and biochemical parameters—serum bilirubin, serum albumin, prothrombin time, ascites and hepatic encephalopathy—into a simple staging system (Child–Pugh classes A, B and C). Owing to its simplicity and long-standing clinical use, it continues to play an important role in routine hepatology practice. Although newer prognostic models such as the Model for End-Stage Liver Disease (MELD) score have increasingly been adopted for organ allocation and mortality prediction, the Child–Pugh classification remains widely utilized in daily clinical practice, particularly among hospitalized patients with decompensated cirrhosis [13,14]. Several studies have demonstrated its value in predicting outcomes across various clinical settings, including portal hypertension, variceal bleeding and liver transplantation. Nevertheless, real-world data evaluating its independent prognostic impact in large cohorts of hospitalized cirrhotic patients remain relatively limited, particularly in tertiary emergency hospital settings where disease severity andcomorbidity burden may differ substantially from those observed in controlled clinical cohorts [15,16,17,18]. Furthermore, the prognostic performance of the Child–Pugh classification with respect to short- and mid-term survival in routine clinical practice warrants further investigation. Large retrospective cohorts with extended observation periods can provide valuable insight into survival differences across disease stages and help clarify the independent contribution of Child–Pugh stage to mortality risk after adjustment for other clinical factors. Therefore, the aim of the present study was to evaluate the Child–Pugh stage as an independent predictor of short- and mid-term survival in a large cohort of patients hospitalized for liver cirrhosis in a tertiary emergency hospital over a 10-year period. By analyzing real-world clinical data, we sought to characterize survival differences across Child–Pugh stages and to identify additional factors associated with mortality in this high-risk population.

Unlike prior comparative studies evaluating Child–Pugh versus MELD, the present study focuses on the independent prognostic value of the Child–Pugh classification in a real-world cohort of hospitalized patients from a tertiary emergency hospital over a 10-year period (2015–2025). This approach highlights its practical applicability in settings where more complex scoring systems may not be readily available. This perspective is particularly relevant, as MELD is not consistently accessible in routine clinical practice in Romania.

## 2. Materials and Methods

### 2.1. Study Design and Population

This retrospective cohort study included patients hospitalized with decompensated liver cirrhosis at our tertiary emergency hospital between 2015 and 2025. A total of 2831 unique patients accounting for 6801 admissions were identified. Among them, 812 had complete clinical data and 631 patients had documented Child–Pugh staging, which was used to assess the prognostic impact of cirrhosis severity.

Laboratory parameters were available for a larger subgroup of patients (*n* = 811) and these data were used for the biochemical analyses.

Child–Pugh staging was missing primarily due to: (a) lack of documentation of the required clinical parameters (hepatic encephalopathy, ascites), particularly in short-term or emergency admissions; (b) interdepartmental transfers without complete clinical documentation; and (c) hospitalizations in which cirrhosis was recorded as a secondary diagnosis and did not undergo a full prognostic evaluation.

### 2.2. Data Collection and Variables

Data were extracted from hospital records and included administrative information, diagnoses, laboratory results, clinical parameters and prognostic scores. Child–Pugh stage was determined according to standard criteria (A, B or C).

“Mixed cirrhosis type” was operationally defined as cirrhosis coded simultaneously as both a primary and a secondary diagnosis during the same hospitalization. This ICD-10 coding pattern was used as a proxy for increased clinical complexity (e.g., multiple etiologies or severe hepatic comorbidity). However, this variable has aninherent administrative component and its results should therefore be interpreted with caution.

Methodological note: Survival was operationally defined as documented event-free time, measured from the first admission to the last recorded discharge or in-hospital death within the available database. Post-discharge mortality data were not available. Therefore, the term “short- and mid-term survival” refers exclusively to documented survival between hospitalizations.

### 2.3. Statistical Analysis

Continuous variables were expressed as mean ± standard deviation or median (interquartile range), depending on data distribution and categorical variables as counts and percentages. Normality was assessed using the Shapiro–Wilk test. Comparisons across Child–Pugh stages were performed using Chi-square tests for categorical variables and Mann–Whitney U or Kruskal–Wallis tests for non-normally distributed continuous variables. Posthoc analyses were adjusted with Bonferroni correction. Associations between ordinal or continuous variables were evaluated using Spearman’s rank correlation coefficient.

Survival analyses were conducted using Kaplan–Meier curves and compared with the log-rank test. Multivariate Cox proportional hazards regression was performed to identify independent predictors of mortality, including age, cirrhosis etiology, laboratory parameters and Child–Pugh stage. Model discrimination was assessed with the C-statistic. A *p*-value < 0.05 was considered statistically significant. Data processing and analysis were performed using Python 3.x (Pandas 3.0.1, NumPy 2.4.0, SciPy 1.17.1, Matplotlib 3.10.0 and Seaborn 0.13 libraries).

The modeling strategy was two-step: (1) a full model including all variables significant in the log-rank analysis; and (2) a final parsimonious model selected using the Akaike Information Criterion (AIC), which retained five independent predictors. The events-per-variable ratio was adequate (EPV = 180 events/5 predictors = 36), exceeding the recommended minimum threshold of 15. Collinearity note: As the Child–Pugh score intrinsically includes ascites and hepatic encephalopathy, the main model excluded these variables as separate covariates when Child–Pugh stage was included.

### 2.4. Ethical Considerations

The study was conducted in accordance with the Declaration of Helsinki and approved by the Institutional Ethics Committee of St. John Emergency Hospital, Approval No. R 3465/Date: 5 March 2026.

This approach allowed the evaluation of the independent prognostic role of Child–Pugh stage on short- and mid-term survival in a real-world emergency hospital setting, while acknowledging that the well-characterized subgroup may not fully represent the entire cohort.

## 3. Results

### 3.1. Baseline Characteristics

Among 2831 patients hospitalized for decompensated cirrhosis between 2015 and 2025, a subgroup of 812 patients had complete clinical data available for analysis. Of these, 631 patients (77.5%) were successfully classified according to the Child–Pugh staging system and comprised the final cohort for survival analysis. The distribution of patients across Child–Pugh stages was 13.4% in Child A (*n* = 85), 31.9% in Child B (*n* = 201), and 54.7% in Child C (*n* = 345). In-hospital mortality increased markedly with worsening liver function, from 1.2% in Child A to 9.0% in Child B, and 46.7% in Child C patients. Median platelet counts showed a decreasing trend across Child–Pugh stages, with 130 × 10^3^/µL in Child A, 131 × 10^3^/µL in Child B, and 112 × 10^3^/µL in Child C. Overall, among the staged patients, 180 deaths occurred, representing 28.5% of the cohort (Table 1). This distribution highlights a clear gradient of disease severity and associated short-term risk, providing the basis for subsequent survival analyses.

### 3.2. Child–Pugh and In-Hospital Mortality

**Figure 1:**
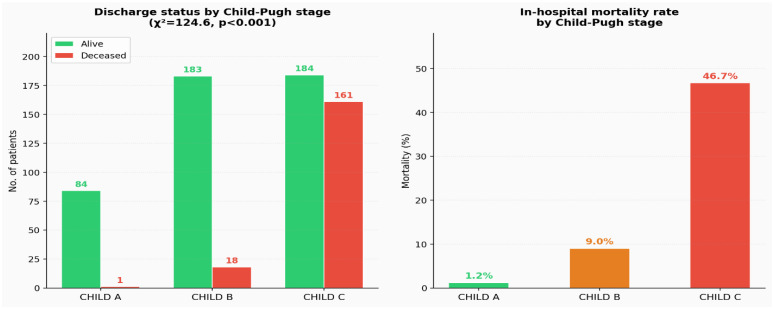
In-hospital mortality and discharge status by Child–Pugh stage.

The association between Child–Pugh stage and in-hospital mortality was evaluated using the Chi-square test. The contingency table comparing discharge status (alive vs. deceased) across Child–Pugh stages showed a statistically significant association (χ^2^ = 124.6, *p* < 0.001), indicating that mortality differed significantly according to disease severity. The distribution of discharge status varied markedly across stages. Among patients classified as Child–Pugh A, 84 out of 85 patients were discharged alive and only one death was recorded. In the Child–Pugh B group, 183 patients survived and 18 died during hospitalization. In contrast, the Child–Pugh C group showed a substantially higher mortality burden, with 184 survivors and 161 deaths, corresponding to a survival-to-death ratio of approximately 1.14:1. In-hospital mortality increased progressively across the three stages (Figure 1), rising from 1.2% in Child–Pugh A to 9.0% in Child–Pugh B and 46.7% in Child–Pugh C. This pattern demonstrates a clear and marked increase in mortality risk with advancing liver disease severity. To facilitate interpretation, the results are illustrated using two complementary graphical representations. The first displays the absolute number of survivors and deaths across Child–Pugh stages, while the second presents the corresponding mortality rates normalized to the number of patients within each stage. Together, these visualizations confirm the strong association between Child–Pugh stage and in-hospital mortality, with a pronounced increase in mortality in patients with advanced disease.

### 3.3. Biochemical Parameters

**Figure 2:**
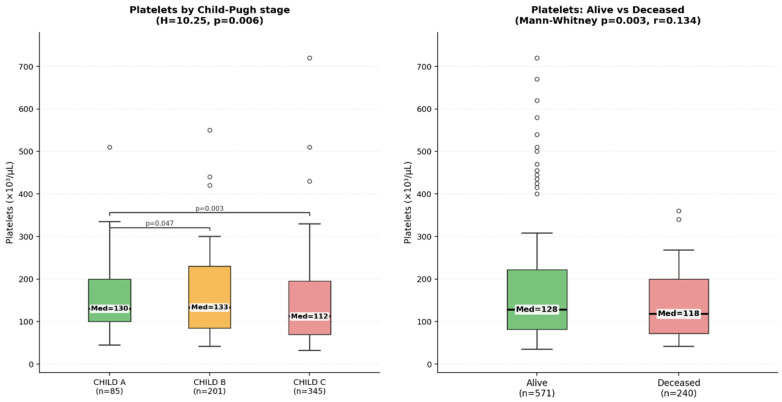
Laboratory parameters associated with mortality. Platelet counts stratified by Child–Pugh stage (**left**) and by discharge status (alive vs. deceased) (**right**).

Biochemical parameters including platelet count, alanine aminotransferase (ALT/TGP) and aspartate aminotransferase (AST/TGO) were analyzed in relation to disease severity and mortality. Platelet counts were available for 811 patients. The mean platelet count was 143,278/µL (SD = 90,025), while the median value was 123,000/µL, suggesting a positively skewed distribution with several high outliers. Therefore, the median value was considered more representative of the central tendency. When stratified by Child–Pugh stage, platelet counts showed a decreasing trend with increasing disease severity. Median platelet values were 130,000/µL in Child–Pugh A patients, 131,000/µL in Child–Pugh B patients and 112,000/µL in Child–Pugh C patients. The overall difference between groups was statistically significant according to the Kruskal–Wallis test (H = 10.25, *p* = 0.006). The lower platelet values observed in Child–Pugh C patients suggest an association between thrombocytopenia and advanced liver disease. Serum aminotransferases also showed wide variability across patients. The median ALT (TGP) level was 26 U/L, with a broad range and several extreme outliers, including values up to 28,100 U/L. The median AST (TGO) level was 47 U/L. An AST/ALT ratio greater than 2, commonly associated with alcohol-related liver disease, was observed in a substantial proportion of patients with alcohol as the primary etiology of cirrhosis. Platelet counts were also analyzed according to discharge status (Figure 2). Patients who died during hospitalization had significantly lower platelet counts compared with those discharged alive (median 118 × 10^3^/µL vs. 128 × 10^3^/µL, *p* = 0.003, Mann–Whitney test), although the magnitude of the difference was modest. Overall mortality in the subgroup with complete biochemical data (*n* = 812) was 29.6% (240/812). Mortality rates were slightly higher in men (30.7%) compared with women (26.4%); however, this difference did not reach statistical significance (*p* = 0.281).

### 3.4. Longitudinal Survival Trends (Kaplan–Meier Analysis)

Kaplan–Meier survival estimates at predefined time points are presented in Table 2.

Overall survival declined progressively during follow-up. Approximately 74.4% of patients survived the first 30 days, while the estimated survival decreased to 49.4% at two years. Clear differences in survival were observed across Child–Pugh stages. Patients classified as Child–Pugh A maintained very high survival throughout the follow-up period, remaining at approximately 98.7%. In contrast, survival decreased progressively in Child–Pugh B patients, from 95.6% at 30 days to 72.4% at two years. The poorest survival was observed in Child–Pugh C patients, where survival declined from 64.6% at 30 days to 28.5% at two years. Log-rank tests confirmed statistically significant differences between survival curves. Comparisons between Child–Pugh A and Child–Pugh C, as well as between Child–Pugh B and Child–Pugh C, were highly significant (*p* < 0.001). The difference between Child–Pugh A and Child–Pugh B was also statistically significant. In contrast, survival estimates for patients with low versus high platelet counts were very similar across all time points, suggesting that platelet count alone did not meaningfully stratify survival outcomes in this cohort.

**Figure 3:**
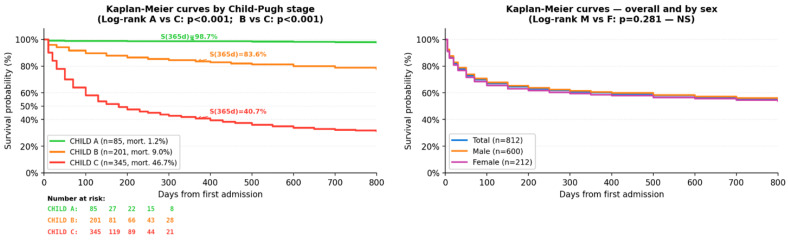
Kaplan–Meier survival curves stratified by Child–Pugh stage (**left**) and by sex (**right**).

The Kaplan–Meier survival curves illustrating these differences are shown in Figure 3. Differences between survival curves were assessed using the log-rank test. Significant differences were observed between Child–Pugh stages (*p* < 0.001).

The Kaplan–Meier curves demonstrate a clear separation between Child–Pugh stages early during follow-up, which remained consistent throughout the observation period. Child–Pugh A patients showed minimal decline in survival, while Child–Pugh B patients demonstrated a gradual decrease over time. Child–Pugh C patients experienced the steepest decline, particularly during the first months of follow-up. When stratified by sex, survival curves for male and female patients showed a similar pattern without visible separation. Log-rank testing confirmed the absence of a statistically significant difference between sexes (*p* = 0.281), indicating that sex was not associated with survival differences in this cohort.

### 3.5. Cox Proportional Hazards Model

#### 3.5.1. Cox Proportional Hazards Regression Analysis

Variables found to be significant in the Kaplan–Meier analysis were further included in a Cox proportional hazards regression model to identify independent predictors of survival. The initial database comprised 6801 hospitalizations corresponding to 2831 unique patients diagnosed with liver cirrhosis between 2015 and 2025. Among these, 812 patients (28.7%) had complete clinical information, including cirrhosis etiology, laboratory parameters, staging data and clinical complications and were included in the main clinical cohort, while the remaining 2019 patients were identified solely through administrative diagnostic codes and analyzed separately as part of an extended administrative cohort. Among the 812 patients with detailed clinical data, 181 (22.3%) lacked complete Child–Pugh staging and were excluded frommultivariable models including this variable, resulting in a final analytic sample of 631 patients. Mortality rates did not differ significantly between patients with and without Child–Pugh staging (33.1% vs. 28.5%; χ^2^ = 1.44, *p* = 0.23), suggesting that missing staging data was not associated with the outcome and supporting the validity of thecomplete-case analysis. Multivariate Cox regression analysis identified severalindependent predictors of mortality (Table 3).

The strongest predictor in the model was the mixed cirrhosis diagnostic category (recorded simultaneously as both primary and secondary diagnosis), which was associated with a markedly increased risk of death (HR = 8.58, 95% CI 4.81–15.32, *p* < 0.001). This category reflects hospitalizations in which cirrhosis was coded simultaneously as both a primary and secondary diagnosis, a pattern that likely captures patients with complex or severe clinical presentations requiring multidisciplinarymanagement. In addition, patients classified as Child–Pugh stage C had a substantially higher mortality risk compared with those in Child–Pugh stage A (HR = 25.11, 95% CI 3.44–183.29, *p* = 0.002). The wide confidence interval observed for this estimate reflects the extremely low number of deaths in the reference group, where only one deathoccurred among 85 Child–Pugh A patients. This limitation introduces statistical instability and reduces the precision of effect size estimation for comparisons involving this group. Therefore, hazard ratio estimates involving Child–Pugh A should be interpreted with caution. Alcohol-related cirrhosis was also independently associated with increased mortality, with an estimated 81% higher risk of death compared with other etiologies afteradjustment for all covariates in the model (HR = 1.81, 95% CI 1.09–3.01, *p* = 0.023). Age showed a modest but statistically significant contribution to mortality risk, with a 17.9% increase in hazard for each standard deviation increase in age at the time of firsthospitalization (HR = 1.18, 95% CI 1.00–1.39, *p* = 0.050). Several additional variables showed non-significant or borderline associations with mortality after adjustment. Child–Pugh stage B demonstrated a non-significant trend toward increased riskcompared with stage A (HR = 6.34, *p* = 0.076), likely reflecting the limited number of deaths in the reference category. Similarly, hepatic encephalopathy (HR = 1.35, *p* = 0.090) and hepatitis B infection (HR = 1.77, *p* = 0.061) showed marginal associations withmortality but did not reach statistical significance. Hepatitis C infection, platelet count and the presence of ascites were not significantly associated with mortality aftermultivariable adjustment, with confidence intervals crossing unity. To evaluate therobustness of the findings, a sensitivity analysis using multiple imputation for missing data (M = 20 datasets, predictive mean matching) was performed and results were pooled according to Rubin’s rules. The imputed analyses yielded hazard ratiosconcordant with the primary complete-case model for 13 of the 14 evaluated variables (92.9%), confirming the stability of the main findings. The statistical power of the model was assessed using the Events Per Variable (EPV) metric. With 180 deaths and fivepredictors included in the parsimonious model, the EPV value was 36.0, substantially exceeding the recommended minimum threshold of 15 and indicating adequatestatistical power for detecting clinically meaningful associations. According to the Schoenfeld formula, the minimum detectable hazard ratio with 80% statistical power (α = 0.05) was estimated at 1.23, which is lower than all significant hazard ratios observed in the model. The proportional hazards assumption was verified using Schoenfeldresiduals and showed no significant violations. Overall model performancedemonstrated good discriminative ability, with a concordance statistic (C-statistic) of approximately 0.80, indicating satisfactory differentiation between patients whosurvived and those who died during follow-up. The final Cox regression analysis was therefore conducted on 631 patients with available Child–Pugh staging, including 180 deaths and 451 survivors within the 2015–2025 study cohort.

#### 3.5.2. Logistic Regression Model

Mortality was modeled as a binary outcome (death, yes/no). Child–Pugh stage was included as an ordinal variable (A = 1, B = 2, C = 3) due to the very low number of deaths in Child A (1/85), which made dummy coding unstable. The model showed excellent discrimination (AUC = 0.865) and explained 64.5% of outcome variance (Nagelkerke R^2^ = 0.645).

**Figure 4:**
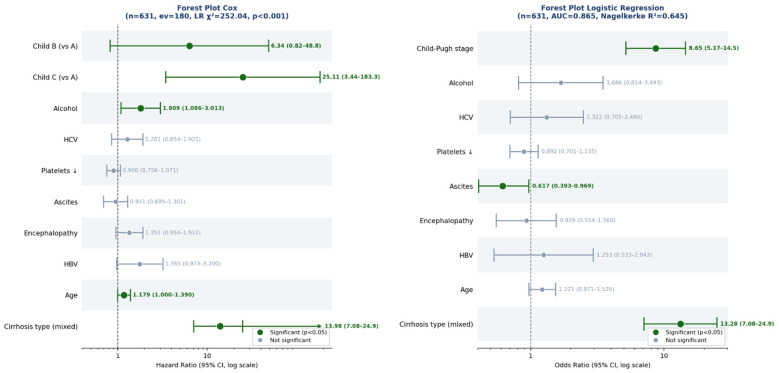
Cox forest plot (**left**): significant predictors were mixed cirrhosis type, Child–Pugh C, alcohol etiology and age. Logistic regression forest plot (**right**): similar pattern, with mixedcirrhosis type and Child–Pugh stage as major predictors, along with ascites as a significant predictor with an inverse association. Legend: Platelets ↓ = Decreased platelets.

The logistic model included nine independent variables (Table 4)**,** of which two reached statistical significance (*p* < 0.05), while the remaining seven did not contribute significantly after adjustment. The overall model was statistically significant (Likelihood Ratio test: χ^2^ = 377.38, df = 9, *p* < 0.001). Among the predictors, mixed cirrhosis type showed the strongest effect (OR = 13.28, *p* < 0.001), indicating that patients with mixed cirrhosis had about13.3-fold higher odds of death compared with those with a primary diagnosis; the 95% CI (7.08–24.90) suggests a relatively stable estimate. Child–Pugh stage was also strongly associated with mortality (OR = 8.66, *p* < 0.001), and because it was coded ordinally (A→B→C), each higher stage increased the odds of death about 8.7-fold (95% CI 5.17–14.49). Ascites was the only significant variable with an OR below 1 (OR = 0.62, *p* = 0.036), indicating an inverse association (about 38% lower odds of death). This counterintuitive result likely reflects confounding, as ascites frequently co-occurs with advanced Child–Pugh stages already controlled in the model. The remaining variables were not statistically significant in the logistic regression model. Alcohol (OR = 1.69, *p* = 0.160) and age (OR = 1.22, *p* = 0.087) showed non-significant trends, although both became significant in the Cox proportional hazards model, suggesting that their effect is more related to the temporal progression of the disease rather than the final outcome alone (Figure 4). Other variables, including HCV (OR = 1.32, *p* = 0.384), thrombocytopenia (OR = 0.89, *p* = 0.353), encephalopathy (OR = 0.93, *p* = 0.782) and HBV (OR = 1.25, *p* = 0.605), were also not significant, with confidence intervals that included value 1.

In the forest plots, each point represents the estimated effect of a predictor, while the horizontal bar corresponds to the 95% confidence interval. When the bar does not intersect the vertical reference line at 1, the association is statistically significant. Points located to the right of the line indicate an increased risk, whereas those to the leftindicate a reduced risk. Narrower confidence intervals reflect greater precision of theestimate. In the Cox model (*n* = 631, events = 180), mixed cirrhosis type emerged as the strongest predictor (HR = 8.58, 95% CI 4.81–15.32, *p* < 0.001), showing the largest hazard ratio with a relatively narrow confidence interval, indicating a stable and robust estimate. Child–Pugh C compared with Child–Pugh A also showed a very large effect (HR = 25.11, 95% CI 3.44–183.29, *p* = 0.002), although the confidence interval was extremely wide, reflecting imprecision due to the very small number of deaths in the Child–Pugh Areference group. Alcohol-related etiology was associated with a moderate but significant increase in risk (HR = 1.81, 95% CI 1.09–3.01, *p* = 0.023). Age showed a small butborderline significant effect (HR = 1.18, 95% CI 1.00–1.39, *p* = 0.050). Other variables, including Child–Pugh B, HCV etiology, thrombocytopenia, ascites, encephalopathy and HBV etiology, had confidence intervals that crossed the reference line, indicating non-significant associations. In the logistic regression model (*n* = 631), mixed cirrhosis type again showed the strongest association with the outcome (OR = 13.28, 95% CI 7.08–24.90, *p* < 0.001), with a clearly right-shifted point estimate and a relatively narrow confidence interval. Child–Pugh stage, coded as an ordinal variable, was also a strong and significant predictor (OR = 8.66, 95% CI 5.17–14.49, *p* < 0.001), providing a more stable estimate than the dummy-coded categories used in the Cox model. Ascites was the only significant predictor with an inverse association (OR = 0.62, 95% CI 0.39–0.97, *p* = 0.036), likely reflecting confounding with the Child–Pugh stage included in the model. Alcohol etiology, HCV, thrombocytopenia, encephalopathy, HBV etiology and age were not statistically significant in the logistic regression model, as their confidence intervals crossed the reference line. Although both models were applied to the same dataset, they produced partially different results. The Cox model identified mixed cirrhosis type, Child–Pugh C, alcohol etiology and age as significant predictors, whereas the logistic model identified mixed cirrhosis type, Child–Pugh stage and ascites. Both models consistently highlighted cirrhosis type as the main predictor. These differences have a methodological explanation: the Cox model incorporates time-to-event information, allowing it to detect effects related to disease progression dynamics, such as those associated with age and alcohol etiology. The logistic model, in contrast, evaluates only the occurrence of the outcome. Nevertheless, its performance was strong, with an AUC of 0.865 indicating excellent discrimination between outcome categories and a Nagelkerke R^2^ of 0.645, suggesting that the model explains approximately 64.5% of the variance in the outcome.

## 4. Discussion

Liver cirrhosis is a major global health burden and represents the end stage of chronic liver disease, leading to significant morbidity and mortality worldwide [1,15,19,20,21]. Decompensated cirrhosis is characterized by the occurrence of seve recomplications such as hepatorenal syndrome, variceal bleeding, hepatic encephalopathy and spontaneous bacterial peritonitis. These events significantly worsen prognosis and are associated with frequent hospitalizations, increased healthcare utilization and reduced survival [22,23,24,25]. Accurate prognostic stratification is essential in these patients to guide clinical decision-making, optimize resource allocation and identify those who may benefit from intensive monitoring or advanced therapeutic interventions. Among the available prognostic tools, the Model for End-Stage Liver Disease (MELD) score is widely used for organ allocation and predicting mortality, while the Child–Pugh classification remains a simple, practical and clinically validated system for bedside risk assessment [14,26,27]. Despite its simplicity, the Child–Pugh score integrates key clinical and laboratory parameters—serum bilirubin, serum albumin, prothrombin time, ascites and hepatic encephalopathy—into a staging system (A, B, C) that effectively predicts outcomes across a range of clinical scenarios, including variceal bleeding, portal hypertension and perioperative risk [28,29,30]. Its ease of use makes it particularly relevant in emergency hospital settings where rapid risk stratification is necessary.

Both the Cox proportional hazards model and the logistic regression analysis identified cirrhosis type and Child–Pugh stage as major predictors of mortality, although some differences were observed in the significance of additional variables such as alcohol-related etiology and age. These differences likely reflect the methodological characteristics of the two models: the Cox regression incorporates time-to-event information, allowing the detection of predictors related to the temporal dynamics of disease progression, whereas logistic regression evaluates only the occurrence of death, regardless of timing. The use of an ordinal representation of Child–Pugh stage in thelogistic regression model provided more stable estimates, partially mitigating the impact of sparse events in the Child–Pugh A reference category observed in the Cox model.

The mortality gradient across Child–Pugh stages was highly non-linear, with aparticularly sharp increase between Child B and Child C stages, as reported in multiple studies evaluating cirrhosis prognosis and the predictive value of Child–Pugh classification [3,15,16,31]. This observation suggests that clinical deterioration associated with advanced cirrhosis may accelerate markedly once patients reach the most severe stage, emphasizing the prognostic relevance of Child–Pugh C classification, as reported in the literature and confirmed by a large Japanese registry study [32]. Separately, interventions such as partial splenic embolization for hypersplenism have been reported to improve hematologic and clinical outcomes in patients with cirrhosis [33,34].

Mixed cirrhosis type showed the strongest statistical association with mortality within the model; however, this finding should be interpreted with caution. As this variable is based on administrative coding (simultaneous primary and secondary diagnosis),it likely reflects increased clinical complexity or documentation patterns rather than a distinct pathophysiological entity. Therefore, this variable should be considered an administrative proxy and not a clinically actionable prognostic factor.

The wide variability observed in aminotransferase levels may reflect acute hepaticinjury episodes or transient elevations during decompensation, highlighting the dynamic nature of liver dysfunction in hospitalized patients. These findings underscore the importance of integrating clinical staging with laboratory markers and potential interventional strategies to enhance risk stratification and guide individualized management. In addition, circulating D-dimers have been investigated as potential prognostic biomarkers in advanced cirrhosis, although their clinical utility remains uncertain [35]. Several limitations should be acknowledged. The retrospective design may introduce selection bias and although Child–Pugh staging was available for a well-characterized subgroup, these findings may not be fully generalizable to all hospitalized patients with cirrhosis. An additional limitation is the lack of important clinical confounders, including treatment-related variables, comorbidities and alternative prognostic scores such as the MELD score. These variables were not consistently available in the retrospective dataset and therefore could not be included in the multivariable models. As a result, residual confounding cannot be excluded and the observed associations should be interpreted within the constraints of available real-world data.

The study focused on in-hospital and inter-admission documented outcomes; post-discharge mortality data were not available, which limits the interpretation of Kaplan–Meier curves and Cox regression analyses as reflecting true overall survival. An important limitation is the substantial attrition from the source cohort (2831 patients) to the final analyzed sample (631 patients, 22.3%), primarily due to incomplete documentation of Child–Pugh staging in the administrative data. This substantial attrition may introduce selection bias, as patients with more complete clinical documentation may differ systematically from the overall hospitalized population.

Excluded patients showed a slightly different clinical profile, which may limit the generalizability of the findings. The inclusion of ascites and hepatic encephalopathy as separate covariates alongside Child–Pugh stage introduces structural collinearity; therefore, the inverse association observed for ascites in the logistic model (OR = 0.62) should be interpreted as an over adjustment artifact.

Nevertheless, the large sample size, extended observation period and use of complementary statistical models strengthen the robustness of the conclusions. These limitations should be interpreted in the context of a real-world dataset, which enhances the clinical relevance and external applicability of the findings despite inherent constraints in data completeness.

## 5. Conclusions

Our study confirms that the Child–Pugh stage remains a strong and independent predictor of short- and mid-term mortality in hospitalized patients with decompensated cirrhosis. Mortality increases sharply from Child–Pugh A to Child–Pugh C, highlighting the score’s practical value for rapid risk stratification in emergency settings. Patients with mixed-type cirrhosis represent a particularly high-risk group, reflecting complex clinical presentations that may require multidisciplinary management. Mixed cirrhosis type reflects an administrative coding pattern (ICD-10 simultaneously coded as primary and secondary diagnosis) and does not represent a standardized clinical entity; the strong statistical association observed should be interpreted with caution and validated prospectively. Complementary analyses using both Cox proportional hazards andlogistic regression models demonstrate that incorporating time-to-event information captures the dynamic progression of disease, while logistic models provide stableestimates of overall mortality risk. The non-linear mortality gradient between Child–Pugh B and Child–Pugh C emphasizes the urgency of early identification andintervention for advanced-stage patients. Integrating clinical staging with laboratory markers and targeted interventions, such as partial splenic embolization, may further enhance prognostic accuracy and guide individualized patient management. These findings should be interpreted in the context of the retrospective study design, missing data, and limited availability of key clinical confounders.

## Figures and Tables

**Table 1:** Baseline characteristics of patients stratified by Child–Pugh stage.

Child–Pugh Stage	Patients Number	% (from 631)	Deaths	Mortality (%)	Median Platelets (×10^3^)
CHILD A	85	13.4%	1	1.2%	130
CHILD B	201	31.9%	18	9.0%	131
CHILD C	345	54.7%	161	46.7%	112
Total staged patients	631	100%	180	28.5%	–

**Table 2:** Kaplan–Meier estimated survival at key follow-up time points.

Time Point	Global S(t)	Child A	Child B	Child C	Low Platelets	High Platelets
30 days	74.40%	98.70%	95.60%	64.60%	70.90%	72.70%
90 days	70.90%	98.70%	93.50%	59.10%	65.70%	71.20%
180 days	67.10%	98.70%	91.00%	54.80%	62.10%	68.00%
1 year (365 d)	57.60%	98.70%	83.60%	40.70%	58.70%	58.60%
2 years (730 d)	49.40%	98.70%	72.40%	28.50%	48.20%	49.00%

**Table 3:** Cox Proportional Hazards Model—Adjusted Hazard Ratios (*n* = 631, events = 180).

Variable	β	HR	95% CI Lower	95% CI Upper	*p*-Value	Clinical Interpretation
Cirrhosis type (mixt vs. primary)	2.150	8.582	4.806	15.323	*p* < 0.001 **	8.6-fold higher risk of death vs. primary cirrhosis
Alcohol-related etiology	0.593	1.809	1.086	3.013	*p* = 0.023 **	Alcohol-related etiology increases the risk of death by 81%
Age (perSD)	0.165	1.179	1.000	1.390	*p* = 0.050 *	17.9% higher risk per SD increase in age
VHC	0.248	1.281	0.854	1.921	*p* = 0.231 NS	Not significant after adjustment
CHILD B (vs. A)	1.846	6.336	0.823	48.776	*p* = 0.076 NS	Not significant trend; extremely wide CI due to one death in the Child–Pugh A reference group.
Decreased Platelets (per SD)	−0.105	0.900	0.756	1.071	*p* = 0.236 NS	Not significant protective trend
CHILD C (vs. A)	3.223	25.113	3.441	183.288	*p* = 0.002 **	25.1-fold higher mortality risk vs. Child–Pugh A; wide CI due to smallreference group (*n* = 85; 1 death)
Encephalopathy	0.301	1.351	0.954	1.912	*p* = 0.090 NS	Marginally non-significant (*p* = 0.09)
VHB	0.568	1.765	0.973	3.200	*p* = 0.061 NS	Marginally non-significant (*p* = 0.06)
Ascites	−0.051	0.951	0.695	1.301	*p* = 0.752 NS	Not significant after adjustment

**Legend:** ** *p* < 0.01; * *p* < 0.05; NS = non-significant.

**Table 4:** Logistic regression—adjusted odds ratios (*n* = 631, events = 180, AUC = 0.865, Nagelkerke R^2^ = 0.645).

Variable	β	OR	95% CI Lower	95% CI Upper	*p*-Value	Clinical Interpretation
Cirrhosis type (mixt vs. primary)	2.586	13.278	7.079	24.903	*p* < 0.001 ***	The strongest predictor in the logistic model—OR of 13.3
Child–Pugh stage (A→B→C)	2.158	8.655	5.169	14.492	*p* < 0.001 ***	Each higher category multiplies the odds of death by 8.7
Ascites	−0.482	0.617	0.393	0.969	*p* = 0.036 *	OR below 1 after adjustment—inverse association (confounding effect)
Alcohol-related etiology	0.522	1.686	0.814	3.493	*p* = 0.160 NS	Not significant in the logistic mode (significant in Cox)
VHC	0.279	1.322	0.705	2.480	*p* = 0.384 NS	Not significant after adjustment
Decreased Platelets (per SD)	−0.114	0.892	0.701	1.135	*p* = 0.353 NS	Not significant protective trend
Encephalopathy	−0.073	0.929	0.554	1.560	*p* = 0.782 NS	Not significant after adjustment
VHB	0.225	1.253	0.533	2.943	*p* = 0.605 NS	Not significant after adjustment
Age (per SD)	0.200	1.221	0.971	1.535	*p* = 0.087 NS	Non-significant trend (*p* = 0.09—marginal NS)

**Legend:** *** *p* < 0.001; * *p* < 0.05; NS = non-significant.

## Data Availability

The raw data supporting the conclusions of this article will be made available by the authors on request due to privacy concerns.
